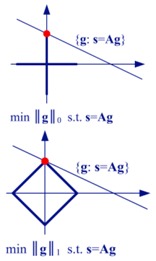# Compressed Sensing Radar Imaging: Fundamentals, Challenges, and Advances

**DOI:** 10.3390/s19143100

**Published:** 2019-07-13

**Authors:** Jungang Yang, Tian Jin, Chao Xiao, Xiaotao Huang

**Affiliations:** College of Electronic Science and Technology, National University of Defense Technology, Changsha 410073, China

**Keywords:** radar imaging, synthetic aperture radar, compressed sensing, sparse reconstruction, regularization

## Abstract

In recent years, sparsity-driven regularization and compressed sensing (CS)-based radar imaging methods have attracted significant attention. This paper provides an introduction to the fundamental concepts of this area. In addition, we will describe both sparsity-driven regularization and CS-based radar imaging methods, along with other approaches in a unified mathematical framework. This will provide readers with a systematic overview of radar imaging theories and methods from a clear mathematical viewpoint. The methods presented in this paper include the minimum variance unbiased estimation, least squares (LS) estimation, Bayesian maximum a posteriori (MAP) estimation, matched filtering, regularization, and CS reconstruction. The characteristics of these methods and their connections are also analyzed. Sparsity-driven regularization and CS based radar imaging methods represent an active research area; there are still many unsolved or open problems, such as the sampling scheme, computational complexity, sparse representation, influence of clutter, and model error compensation. We will summarize the challenges as well as recent advances related to these issues.

## 1. Introduction

**Figure 1:**
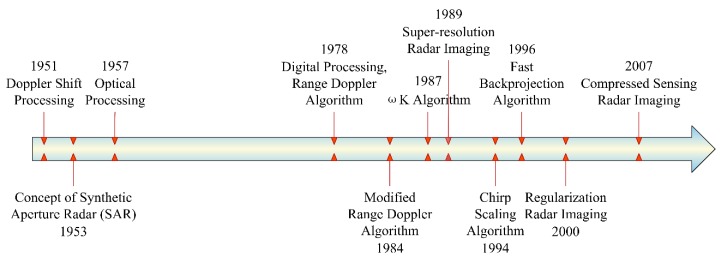
Developmental history of radar imaging methods.

Radar imaging technique goes back to at least the 1950s. In the past 60 years, it has been stimulated by hardware performance, imaging theories, and signal processing technologies. Figure 1 shows the developmental history of radar imaging methods.

Since the development of radar imaging techniques, the main theory that has been used has always been matched filtering [1,2,3]. Matched filtering is a linear process; it has the advantages of simplicity and stability. However, the drawbacks of the matched filtering method are also obvious. Since it does not exploit any prior information concerning the expected targets, its performance is limited by the signal bandwidth. It also requires a dense sampling to record the signals, according to the Shannon–Nyquist sampling theorem. Thus, the matched filtering method places significant requirements on the measured data, but only produces results with limited performance. As higher and higher imaging performance is demanded, the matched filtering method will struggle to meet the requirements.

Apart from the matched filtering framework, from a more generic mathematical viewpoint, radar imaging can be viewed as an inverse problem [4,5,6,7], whereby a spatial map of the scene is recovered using the measurements of the scattered electric field. The radar observation process is a Fredholm integral (F-I) equation of the first kind [8]. Due to observation limitations, such as limited bandwidth and limited observation angles, this inverse problem is usually ill-posed [9,10]. The classic least squares (LS) estimation method cannot solve such ill-posed inverse problems efficiently. The matched filtering method can be viewed as using an approximation to eliminate the irreversible or unstable term in the LS solution. This approximation leads to limited resolution and side-lobes in the results. Thus, matched filtering methods typically provide an image that blurs the details of the scene. Using proper models for the targets, super-resolution methods can improve the resolution of the imaging result [11,12].

Besides using approximation, the ill-posed inverse problem can be solved by another approach, i.e., adding an extra constraint to the LS formula and yielding a stable solution. This approach is called regularization [8]. In order to make the solution after regularization closer to the true value, the additional constraint should represent appropriately some prior knowledge. The regularization approach can also be explained by the Bayesian maximum a posteriori (MAP) estimation theory [6,13,14], which uses prior knowledge in a probabilistic way.

In the radar imaging scenario, imposing sparsity is one possible form of prior knowledge [15]. The advantages of the sparsity-driven regularization methods include increased image quality and robustness to limitations in data quantity. Compressed sensing (CS) refers to the use of under-sampled measurements to obtain the coefficients of a sparse expansion [16,17,18,19,20].

This paper summarizes the fundamentals, challenges and recent advances of sparse regularization and CS-based radar imaging methods. Using a unified mathematical model, we derive the best estimator (i.e., the minimum variance unbiased estimator), the LS estimator, the Bayesian MAP estimator, matched filtering, regularization, and CS reconstructions of the scene. The characteristics of these methods and their connections are also analyzed. Finally, we present some key challenges and recent advances in this area. These include the sampling scheme, the computational complexity, the sparse representation, the influence of clutter, and the model error compensation.

## 2. Mathematical Fundamentals of Radar Imaging

### 2.1. Radar Observation Model

In the continuous signal domain, under the Born approximation, the radar observation process can be denoted as [4]

(1) $$s(r)=\int A(r,r^{\prime })g(r^{\prime })dr^{\prime }+n$$

where s(r) denotes the observed data at the observation position of r, $g(r^{\prime })$ denotes the reflectivity coefficient at $r^{\prime }$ in the scene, $A(r,r^{\prime })$ denotes the system response from $r^{\prime }$ to r, and n denotes noise.

Assuming the system is shift invariant, Equation (1) can be rewritten as

(2) $$s(r)=\int A(r-r^{\prime })g(r^{\prime })dr^{\prime }+n$$

It can be seen that the radar observation model is a convolution process. Equation (1) is a Fredholm integral (F-I) equation of the first kind [8]. From a mathematical viewpoint, radar imaging can be viewed as the solution of the F-I equation—i.e., we want to recover g(r) from the observed data s(r) using the observation equation. Unfortunately, according to the theory of integral equations, solving the F-I equation is usually an ill-posed problem [8].

In practice, since digitization is commonly used, the observed data are discrete. Based on Equation (1), the discrete observation model can be written as

(3) s=Ag+n

where s is stacked from the samples of s(r), g is stacked from the samples of $g(r^{\prime })$, A is formed from samples of $A(r,r^{\prime })$, and n is the observation noise vector.

### 2.2. Best Linear Unbiased Estimate and Least Squares Estimate of the Scene

From the observation model shown in (3), radar imaging can be viewed as an estimation problem, in which the scene g is estimated based on the observed data s in a noisy environment. According to estimation theories, the minimum variance unbiased estimate is the “best” estimate in terms of estimation square error. From Equation (3), it can be seen that when the radar observation model is linear, the minimum variance unbiased estimate is the best linear unbiased estimate [13]—i.e., the expression of the best estimate of the scene is

(4) $$\hat{g}={\left(A^{H}C^{-1}A\right)}^{-1}A^{H}C^{-1}s$$

where C is the covariance matrix of the noise term ($C=E\left[nn^{H}\right]$).

In practice, a more tractable approach is LS estimation, which can be denoted as

(5) $$\hat{g}=\arg\underset{g}{\min}\;{\left\|s\text{-}Ag\right\|}_{2}^{2}$$

Therefore, the LS estimate of the scene is

(6) $$\hat{g}={\left(A^{H}A\right)}^{-1}A^{H}s$$

If n is white Gaussian noise, we have $C=\sigma^{2}I$, where I is the identity matrix. Under such condition, Equations (4) and (6) are the same. Therefore, the LS estimate will equal to the best estimate in white Gaussian noise [13].

If we want to use Equation (6) to calculate the best estimate of the scene, a prerequisite is that $\left(A^{H}A\right)$ is invertible. However, in practice, this prerequisite is usually not satisfied, as discussed below. We assume that the size of A is M×N, where M denotes the number of measurements and N denotes the number of unknown grid points. Then, the size of $\left(A^{H}A\right)$ is N×N.

One case is that M<N, i.e., the number of measurements is less than the unknown variables. CS is a typical example of this case. In such a case, $\mathrm{rank}(A^{H}A)=\mathrm{rank}(A)\leq M<N$, i.e., $\left(A^{H}A\right)$ is irreversible.

In the above case, it can be seen that due to limited number of measurements, $\left(A^{H}A\right)$ is irreversible. Is it possible to make $\left(A^{H}A\right)$ invertible by increasing the number of measurements (i.e., make M>N. As mentioned previously, due to physical limitations, such as limited bandwidth and limited observation angles, if we take more measurements, the interval between the adjacent measurements will be smaller. Thus, the coherence between the adjacent columns in A will increase. Consequently, ${\left(A^{H}A\right)}^{-1}$ will probably be ill-conditioned.

In summary, the LS solution usually contains irreversible or ill-posed terms. This problem is inherent, and is derived from the property of the F-I equation of the first kind [8].

### 2.3. Matched Filtering Method

Examining Equation (6), it can be seen that the irreversible or ill-posed term is ${\left(A^{H}A\right)}^{-1}$. We can multiply $\left(A^{H}A\right)$ in the left side of Equation (6) to eliminate ${\left(A^{H}A\right)}^{-1}$. In this way, we can avoid explicitly calculating the nonexistent or unstable term ${\left(A^{H}A\right)}^{-1}$. This leads to the matched filtering method, which can be denoted as

(7) $${\hat{g}}_{\mathrm{MF}}^{}=(A^{H}A)\hat{g}=A^{H}s$$

Equation (7) can be viewed as multiplying the best estimate of the scene with $\left(A^{H}A\right)$. The matrix $\left(A^{H}A\right)$ is the autocorrelation of the system response, which usually has a sinc pulse shape [1,21]. The matched filtering result can be viewed as the convolution of the best estimate of the scene and the sinc function. A point target will be spread, and side-lobes will also appear in the matched filtering result [21]. This implies that the matched filtering method can only provide an image that blurs the details of the scene. The matched filtering method has a limited resolution, which depends on the autocorrelation of the system response [1].

**Figure 2:**
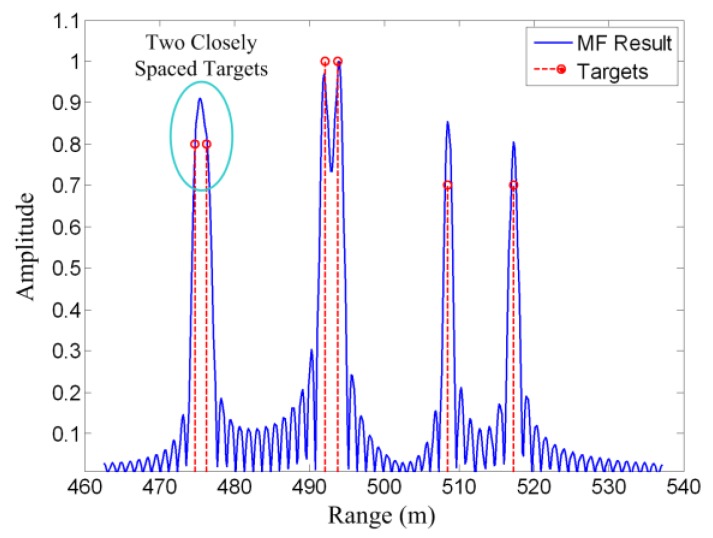
Matched filtering example. Two closely spaced targets cannot be resolved.

Figure 2 shows an example of the matched filtering method. Six point targets are set in the scene. It can be seen that the matched filtering result is the convolution of the targets and the autocorrelation of the system response. As a result, an idea point target is spread into a sinc waveform. Consequently, targets will interfere with each other, and two closely spaced targets may not be resolved in the matched filtering result.

Equation (7) is the original form of the matched filtering equation. In practice, in order to reduce the computational cost and make it more convenient for implementation, some transformations and approximations are usually adopted for Equation (7). Equation (7) can represent many widely used imaging algorithms, such as backprojection algorithms, range Doppler algorithms, chirp scaling algorithms, and ωK algorithms [1].

### 2.4. Regularization Method

Examining the LS formula (Equation (5)), it can be seen that it only relies on the observed data. In order to make the ill-posed inverse problem become well-posed, we can add an extra constraint to the LS formula [8,9,10]. This leads to the regularization method, which can be denoted as

(8) $$\hat{g}=\arg\underset{g}{\min}\;\{{\left\|s\text{-}Ag\right\|}_{2}^{2}+\lambda L(g)\}$$

where λ is the regularization parameter and L(g) is the added penalty function. In order to make the solution of Equation (8) closer to the true value, L(g) should represent appropriate prior knowledge for the problem.

A typical choice of L(g) is

(9) $$L(g)={\left\|g\right\|}_{p}^{p}$$

where ${\left\|\cdot \right\|}_{p}$ denotes the $l_{p}$-norm, i.e.,

(10) $${\left\|g\right\|}_{p}=\left\{ \begin{array}{l} {\left(\sum_{i=1}^{N}{\left|g_{i}\right|}^{p}\right)}^{1/p}\;p>0\\ \mathrm{Number}\;\mathrm{of}\;\mathrm{nonzero}\;\mathrm{elements}\;\mathrm{in}\;g\;p=0\; \end{array}\right.$$

Then, Equation (8) can be rewritten as

(11) $$\hat{g}=\arg\underset{g}{\min}\;\{{\left\|s\text{-}Ag\right\|}_{2}^{2}+\lambda {\left\|g\right\|}_{p}^{p}\}$$

The choice of p can control the result of the regularization method. If we want to enforce sparsity in the result, we should choose p in the range 0≤p≤1 [16,17]. For p=1, Equation (11) can be compared to the Lasso solution of the CS type methods [16]. Equation (11) can be solved by gradient search algorithms, such as the Newton iteration [22].

### 2.5. Bayesian Maximum a Posteriori Estimation

It should be noted that in Equation (11), the added constraint term $\lambda {\left\|g\right\|}_{p}^{p}$ represents prior knowledge [17,23]. Another prior knowledge-based estimation method is Bayes theory. The main idea behind the Bayesian estimation framework is to account explicitly for the errors, and also for incomplete prior knowledge. Assuming that the noise n in Equation (3) is white and Gaussian, we have

(12) $$p(n)\propto \exp\left\{-\frac{1}{2\sigma^{2}}{\left\|n\right\|}_{2}^{2}\right\}$$

where $\sigma^{2}$ is the noise variance. Then we obtain the expression of likelihood

(13) $$p(s|g)\propto \exp\left\{-\frac{1}{2\sigma^{2}}{\left\|s-g\right\|}_{2}^{2}\right\}$$

We assume that the scene has a prior probability density function, as

(14) $$p(g)\propto \exp\left\{-\alpha {\left\|g\right\|}_{p}^{p}\right\}$$

If 0≤p≤1, the magnitude of the scene is more likely to concentrate around zero, which implies that the scene is sparse. For a review on sparsity enforcing priors for the Bayesian estimation approach, the reader can refer to [6].

Using the prior probability density of g shown in (14), and according to the Bayes rule, we obtain

(15) $$p(g|s)=\frac{p(s|g)p(g)}{p(s)}\propto \frac{1}{p(s)}\exp\left\{-\frac{1}{2\sigma^{2}}{\left\|s-g\right\|}_{2}^{2}-\alpha {\left\|g\right\|}_{p}^{p}\right\}$$

Then the MAP estimate can be obtained easily as

(16) $$\hat{g}=\arg\underset{g}{\max}p(g|s)=\arg\underset{g}{\min}\;{\left\|s-g\right\|}_{2}^{2}+2\sigma^{2}\alpha {\left\|g\right\|}_{p}^{p}$$

Comparing Equations (11) and (16), it can be seen that when $\lambda =2\sigma^{2}\alpha$, these two equations are equivalent, i.e., the regularization method is equivalent to Bayesian MAP estimation.

### 2.6. Compressed Sensing Method

For the observation model shown in Equation (3), if the scene (i.e., g) is sparse, according to CS theory, it can be stably reconstructed using reduced data samples. The reconstruction method can be written as [16,17]

(17) $$\hat{g}=\arg\underset{g}{\min}\;{\left\|g\right\|}_{0}\;s.t.\;{\left\|s\text{-}Ag\right\|}_{2}^{2}<\varepsilon$$

where s.t. means subject to and ε denotes the allowed data error in the reconstruction process.

Equation (17) is NP-hard and computationally difficult to solve [17]. Matching pursuit is an approximate method for obtaining an $l_{0}$ sparse solution. In CS theory, a more tractable approach is taking the $l_{1}$-norm instead of the $l_{0}$-norm, which is called the $l_{1}$ relaxation:

(18) $$\hat{g}=\arg\underset{g}{\min}\;{\left\|g\right\|}_{1}\;s.t.\;{\left\|s\text{-}Ag\right\|}_{2}^{2}<\varepsilon$$

If g is sparse and A satisfies some specific conditions, Equations (18) and (17) will have the same solution, and this solution is the exact or approximate recovery of g [16,17]. Equation (18) can be solved using convex programming, which is more tractable than the original $l_{0}$-norm minimum problem. Unlike the matched filtering method, CS method does not have an exact or pre-defined resolution, since it is a non-linear method. Generally, the resolution capability of the CS method is much better than the matched filtering method if the targets are sparse.

**Figure 3:**
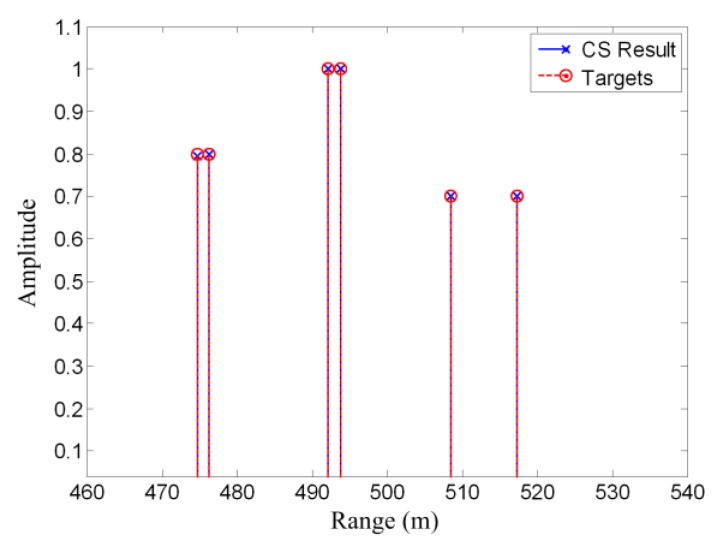
Compressed sensing example; closely spaced targets are well resolved.

Figure 3 shows an example of compressed sensing. The simulated scene is the same as the matched filtering example shown in Figure 2. Only 1/20 signal samples are used for the CS reconstruction. It can be seen that the two closely spaced targets are well resolved. This implies that the CS method can obtain better results using less data than the matched filtering method. The reason is that prior information concerning signal sparsity is utilized in the CS model.

Equation (18) is a constrained optimization problem. According to the Lagrange theory, it can be transformed into an unconstrained optimization problem, which will have the same form as Equation (11). For appropriate choices of λ and p=1, Equations (11) and (18) will be equivalent [16,17]. This implies that CS is a special case of the regularization method.

### 2.7. Summary of Radar Imaging Methods

The above subsections introduced the LS estimator, matched filtering, regularization methods, Bayesian MAP estimation, and the CS method. In this subsection, we will summarize these methods and analyze their connections.

Table 1 lists the main characteristics and describes some connections between these imaging methods. The LS estimation only relies on the observed data, and cannot solve the ill-posed radar imaging problem efficiently. The matched filtering method can be viewed as using an approximation to avoid the ill-posed term in the LS solution. The regularization method, Bayesian MAP estimation, and the CS method exploit prior knowledge concerning the targets in addition to the observed data, and they are equivalent in some cases.

Table 1 also shows the equivalent geometric illustration for each method in ${\mathbb{R}}^{2}$. The observation equation can only confine the solution to a hyperplane (which becomes a line in ${\mathbb{R}}^{2}$), but cannot reliably produce a certain solution [17,23]. The other methods aim at obtaining a stable solution close to the true value, using some modifications that represent prior knowledge concerning the targets.

**Figure 4:**
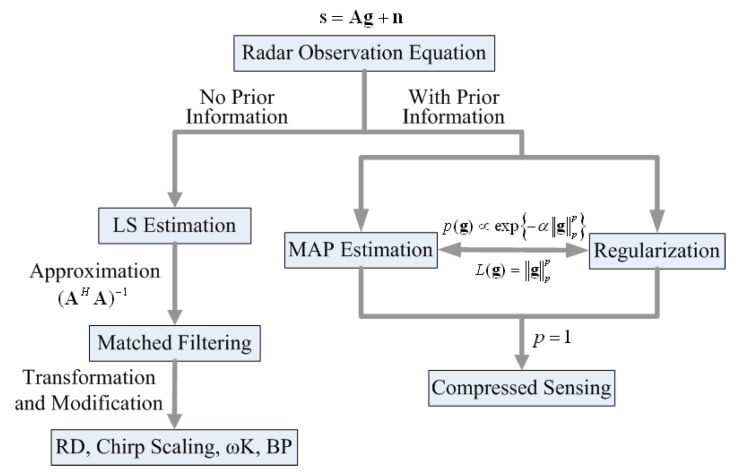
Block diagram and relationship of the radar imaging methods.

Figure 4 shows the block diagram and the relationship of the radar imaging methods. All of the radar imaging methods can be divided into two branches. The first branch does not use the prior information of the targets or scene, and it leads to the linear imaging methods; the most typical and widely used one in this branch is matched filtering. Another branch uses the prior information of the targets or scene. This leads to the non-linear methods. The most recently developed methods, including regularization methods, Bayesian methods, and CS methods belong to this branch.

## 3. Challenges and Advances in Compressed Sensing-Based Radar Imaging

The use of regularization methods in radar imaging goes back at least to the year 2000 [21,24]. Since the CS theory was proposed in 2006, it has been explored for a wide range of radar [25,26,27,28,29,30,31,32,33] and radar imaging applications [4,34,35,36,37,38], including synthetic aperture radar (SAR) [39,40,41,42], inverse SAR (ISAR) [43,44,45], tomographic SAR [46,47,48,49,50,51], three-dimensional (3D) SAR [52,53,54], SAR ground moving target indication (SAR/GMTI) [55,56,57,58,59,60,61], ground penetrating radar (GPR) [62,63,64], and through-the-wall radar (TWR) [65,66,67]. In this paper, we will focus on two-dimensional (2D) imaging radar systems, i.e., SAR, GPR, and TWR.

After several years of development, although many interesting ideas have been presented in this area, there still exist a number of challenges, both in theory and practice [68]. The state of the art in this area has not yet reached the stage of practical application. We will present some challenges as well as recent advances in this part of the paper.

### 3.1. Sampling Scheme

CS usually involves random under-sampling [16,17]. A widely used waveform in traditional radar imaging is the linear frequency modulated (LFM) waveform. If we adopt the LFM waveform in CS-based radar imaging, a random sampling analog to digital (A/D) converter is needed, which is not easily realized in practice. This will require extra hardware components, which means that LFM waveforms are not ideally suited for CS.

Recently, many researchers have found that the stepped frequency waveform is much more suitable for CS than the LFM waveform [35,62,63,66,69]. Sparse and discrete frequencies are more convenient for hardware implementation. For a CS-based radar imaging system, a stepped frequency waveform may be the preferred choice. In practical application, a set of adjustable pseudorandom numbers can be generated to select the frequency points in the stepped frequencies. In this way, randomly generated frequencies, i.e., random and sparse measurement, can be realized, and the CS-based imaging model can be implemented.

**Figure 5:**
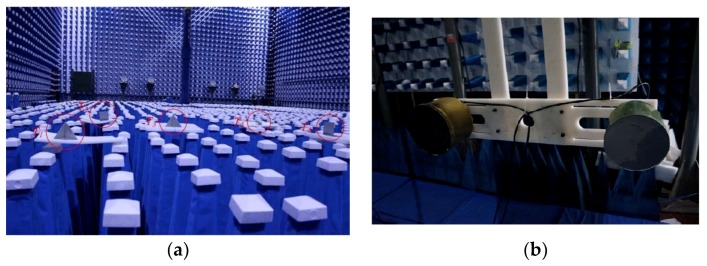
Experimental scene for CS-based stepped frequency radar imaging. (**a**) Five reflectors in the microwave chamber. (**b**) Transmitter and receiver antennas.

**Figure 6:**
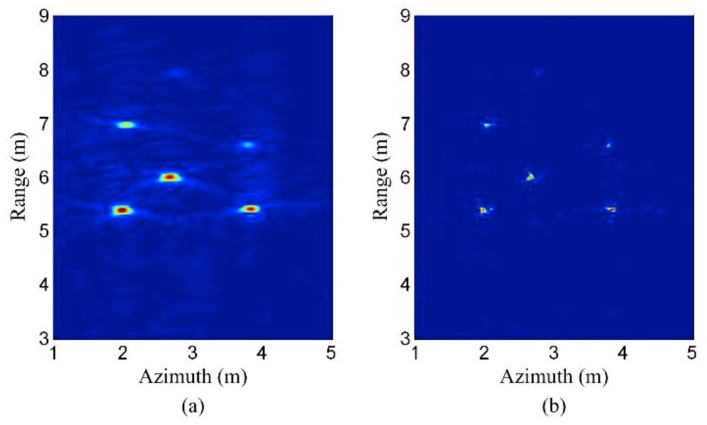
(**a**) Backprojection result of full data (81 azimuth measurements × 2001 frequencies). (**b**) CS result of under-sampled data (27 azimuth measurements × 128 frequencies).

Figure 5 and Figure 6 show an example for CS-based stepped frequency radar imaging. The main equipment in the experimental system is a vector network analyzer (VNA). The experiment is carried out in a non-reflective microwave chamber. Five targets in the scene are shown in Figure 5. Figure 6a shows the backprojection result, using the fully sampled data (81 azimuth measurements × 2001 frequencies). Figure 6b shows the CS reconstruction result using under-sampled data (27 azimuth measurements × 128 frequencies). Considering the aspects of resolution and sidelobe levels, the CS reconstruction result is even better than the backprojection result, although it uses less sampled data. The reason is that prior information concerning signal sparsity is used in the CS model, while the backprojection method uses no prior information.

### 3.2. Computational Complexity

In the regularization or CS model for a 2D radar imaging system, the 2D observed data and the 2D scene grid are both stacked into column vectors. This will lead to a huge size measurement matrix. For example, the original fully sampled data are 2048 × 2540 points (azimuth × range); if a 512 × 512 pixel image is reconstructed from a reduced sampling data consist of 256 × 256 points. Then the size of the matrix A is 65,536 × 262,144. Since regularization or CS reconstruction is a non-linear process, such a large measurement matrix will result in a huge computational burden for image reconstruction. In addition, the total memory to access the measurement matrix is 128 gigabytes (assuming float point and complex numbers are used). This is a too much memory space for normal desktop computers. Considering that data size is usually larger than the above example in practice, it is difficult for conventional methods to reconstruct a moderate-size scene by using normal computers.

**Figure 7:**
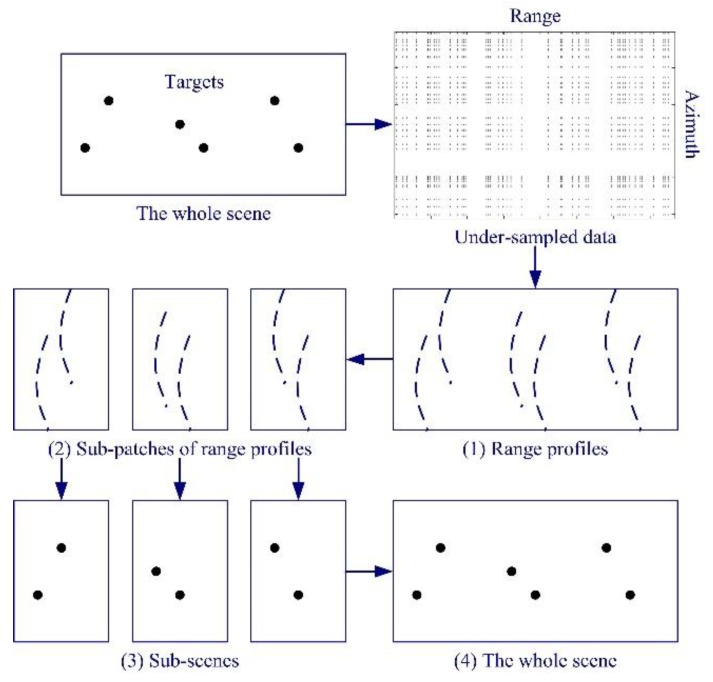
Processing steps of the segmented reconstruction method for CS-based synthetic aperture radar (SAR) imaging (taken from [71]).

A common idea for reducing computational complexity and memory occupancy is to split big data into sets of small data [70]. Based on this thought, a segmented reconstruction method for CS based SAR imaging has been proposed [71]. In this method, the whole scene is split into a set of small subscenes. Since the computational complexity is non-linear to the data size, the reconstruction time can be reduced significantly. The sensing matrices for the method proposed in [71] are much smaller than those for the conventional method. Therefore, the method also needs much less memory. Due to the short reconstruction time and lower memory requirement of the method proposed in [71], reconstructing a moderate-size scene in a short time is no longer a difficult task. The processing steps of the segmented reconstruction method are shown in Figure 7.

**Figure 8:**
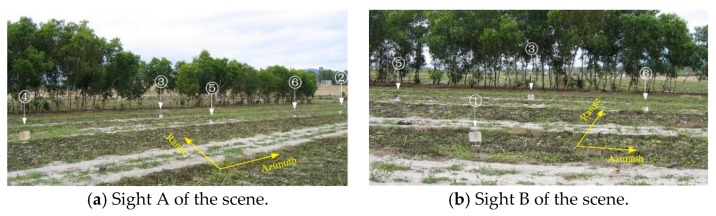
Trihedral reflectors in the scene. Trihedral reflectors 1–4 are large, and trihedral reflectors 5 and 6 are small (taken from [71]).

**Figure 9:**
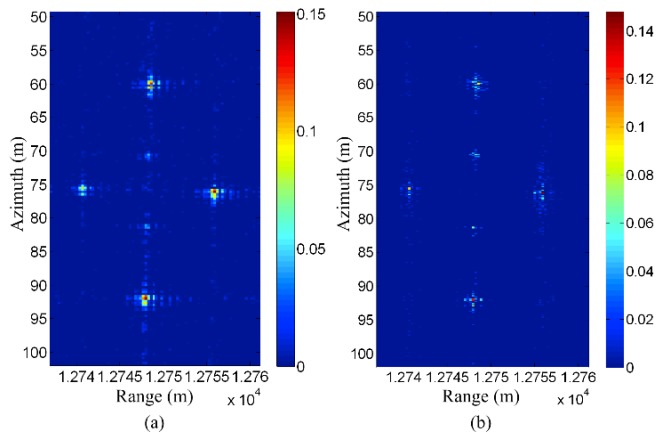
(**a**) Conventional CS reconstruction result (reconstruction time = 44,032 s). (**b**) Segmented reconstruction result (reconstruction time = 1498 s) (taken from [71]).

Figure 8 and Figure 9 show an example of the segmented reconstruction method [71]. Figure 8 shows the experimental scene of an airborne SAR system, which contains six trihedral reflectors. Figure 9a shows the conventional CS reconstruction result, where the reconstruction time is 44,032 s (12 h 14 min). The whole scene is split into five segments, and Figure 9b shows the segmented reconstruction result, where the reconstruction time is now reduced to 1498 s (25 min). It can be seen that, using the segmented reconstruction method, the reconstruction time is significantly reduced, while the reconstruction precision is nearly the same.

### 3.3. Sparsity and Sparse Representation

Sparsity of the scene is an essential requirement for sparse regularization or CS methods. For an SAR scene, an extended scene is usually not sparse in itself (not sparse in the canonical basis), except for the case of a few dominant scatterers in a low reflective background [35]. Therefore, a sparse representation is needed to use a sparsity-driven method.

CS-based optical imaging has successfully used sparse representations [72]. However, radar imaging involves complex-valued quantities; the raw data and the imaging result are both complex-valued. Since the phase of the scene are potentially random, it is very difficult to find a transform basis to sparsify a complex-valued and extended scene [73,74].

Structured dictionaries and dictionary learning ideas are proposed in [75] and [76], respectively. An alternative approach is to handle the magnitude and phase separately [41]. Although the phase of the scene is potentially random, the magnitude of the scene usually has better sparse characteristics. However, this approach has a much higher computational complexity than standard CS reconstruction. Another method investigates physical scattering behavior [4,77]. For example, a car can be represented as the superposition of responses from plate and dihedral shapes.

**Figure 10:**
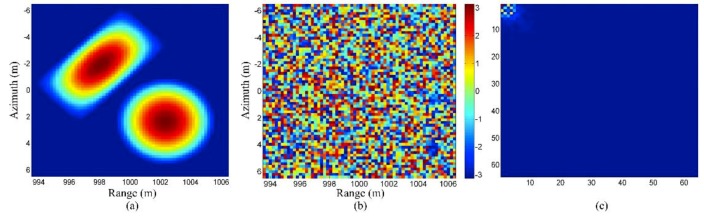
(**a**) Magnitude of the scene, (**b**) phase of the scene, (**c**) DCT result of the magnitude (taken from [79]).

Figure 10 shows a simulation example for an extended and complex-valued scene. There are two extended objects in the scene, one of which has a round shape while the other has a rectangular shape. Both the two objects have random phases associated with them. It can be seen that the DCT (Discrete Cosine Transform) results of the magnitude are sparse.

**Figure 11:**
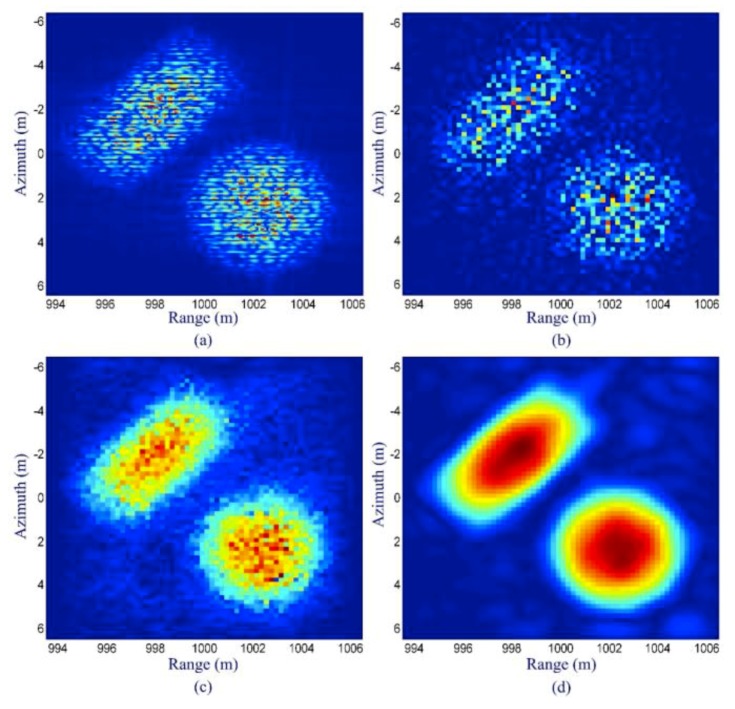
Simulation results: (**a**) matched filtering result, (**b**) conventional CS reconstruction result without sparse representation, (**c**) result of the method with magnitude sparse representation, and (**d**) result of the method with improved magnitude sparse representation (taken from [79]).

Figure 11a shows the result of matched filtering. Since the random phase leads to speckle, it can be seen that although the scene has a smooth shape, the matched filtering result has obvious fluctuation. Figure 11b shows the result of conventional CS reconstruction without sparse representation. The reconstruction algorithm is SPGL1 [78]. Since the scene is not sparse in the canonical basis, the reconstruction is not accurate. Figure 11c shows the result of the method using a magnitude sparse representation [41]; it can be seen that the reconstruction result is much better than Figure 11a,b. Figure 11d shows the result of the method using the improved magnitude sparse representation method proposed in [79]. In the proposed method, besides the sparsity, the real-valued information of the magnitude and the coefficient distribution of the sparse representation are also utilized. It can be seen that both the shape and speckle are further improved.

**Figure 12:**
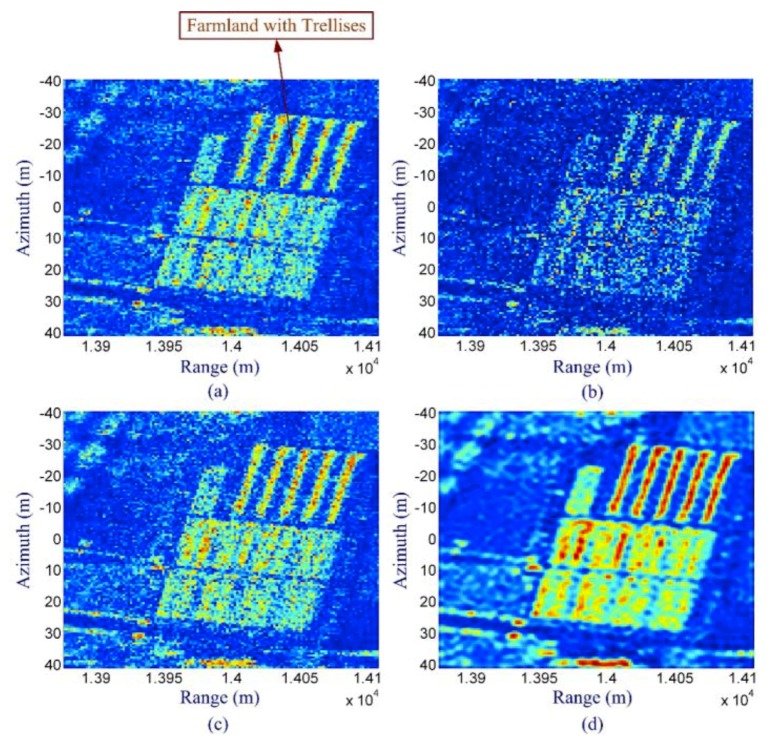
Real data reconstruction results (scene of farmland with trellises): (**a**) matched filtering result (full data), (**b**) conventional CS reconstruction result without sparse representation, (**c**) result of CS with magnitude sparse representation, and (**d**) result of CS with improved magnitude sparse representation (taken from [79]).

Figure 12 shows the real data results. The raw data is acquired by an airborne SAR system. Figure 12 contains a scene of farmland with trellises. The reflectivity from the trellises is very strong. From the real data result, it can be seen that CS with the improved magnitude sparse representation method can produce an image with less speckle and clearer edges of different regions than the previous methods.

### 3.4. Influence of Clutter

Another practical case is when the targets of interest are sparse, but there also exists clutter in the scene. Clutter arises from reflections within the scene, so the image may no longer be sparse if significant clutter returns are present. Typical examples include GPR and TWR imaging. The interesting targets, such as landmines and humans, are usually sparse, but they are often buried in the ground surface clutter and wall clutter.

Some methods have been proposed to remove the ground surface clutter and wall clutter for downward-looking GPR and TWR [64,65]. These methods are effective in cases when the clutter is concentrated in a fixed range cell or limited to several range cells.

Another scenario is TWR/SAR imaging of moving targets. A sparsity-driven change detection method is proposed in [67]. The stationary targets and clutter are removed via change detection, and then CS reconstruction is applied to the resulting sparse scene. In [55], a SAR/GMTI method using distributed CS is proposed, which can cope with the non-sparse stationary clutter.

**Figure 13:**
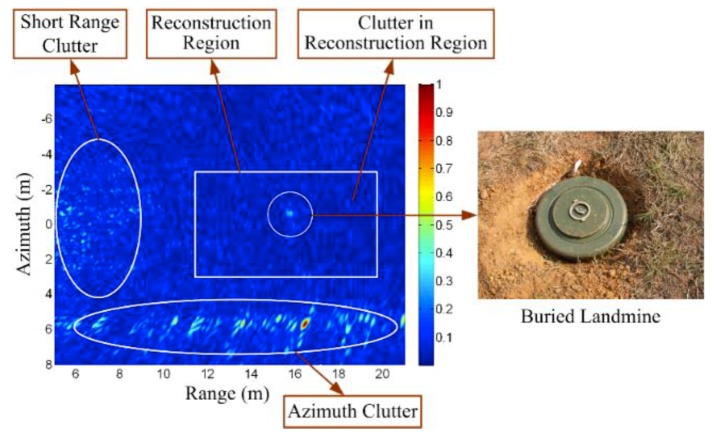
Real data example for the clutter problem in forward-looking GPR (backprojection result using full-sampled data). Taken from [80].

**Figure 14:**
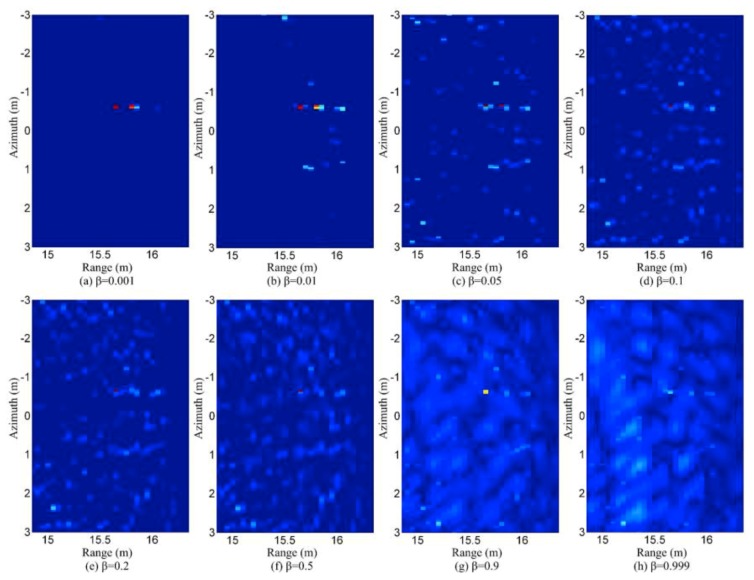
Reconstruction results in clutter environment with different parameters (taken from [80]).

A more difficult case is when both the targets and clutter are stationary, and the clutter is distributed over the whole scene. Forward-looking GPR may fall into this category. Figure 13 shows a real data example for this case. In such a scenario, shrubs and rocks above the ground surface may cause strong azimuth clutter. Short range clutter is usually also strong, due to the large grazing angle and short range. Besides the strong clutter far away from the target (landmine), there is also ground surface clutter around the target. In [68], an idea is proposed to build a model in which the clutter is also taken into account as a norm in the objective function. In [80], the forward-looking clutter is suppressed in two steps. In the first step, the strong clutter outside of the reconstruction region is suppressed first. In the second step, the clutter in the reconstruction region is suppressed by selecting a proper β, which represents the ratio of the non-zeros area in the reconstructed scene. The reconstruction results are shown in Figure 14.

### 3.5. Model Error Compensation

**Figure 15:**
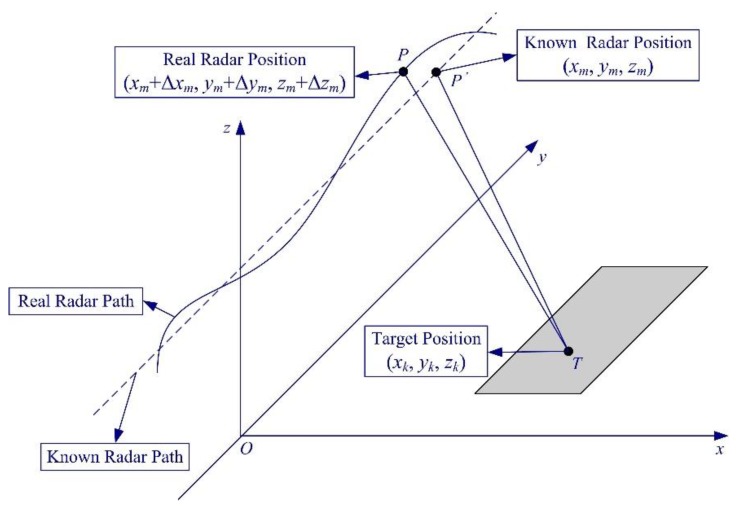
Geometry of the observation position errors in SAR. (Taken from [84]).

In the regularization or CS methods, we usually assume that the model is exact. However, in practice, the model may also contain errors. For example, imperfect knowledge of the observation position will lead to errors in the measurement matrix. This effect resembles motion errors that arise in traditional airborne SAR imaging. Figure 15 shows the geometry of the observation position errors or motion errors in SAR.

Several methods have been proposed to deal with model errors in CS-based or sparsity-driven radar imaging. A phase error correction method for sparsity-driven SAR imaging is proposed in [81]. An autofocus method for compressively sampled SAR is proposed in [82]. This method can correct phase errors in the reconstruction process. Both the methods proposed in [81,82] deal with phase errors in the observed data, or approximately treat the observation position-induced model errors as phase errors in the observed data. In [83], the platform position errors are investigated and compensated. That method considers the azimuth offset errors and also uses some approximations.

In [84], a model error compensation method is proposed. An iterative algorithm cycles through steps of target reconstruction, and observation position error estimation and compensation are used. This method can estimate the observation position error exactly, while only relying on the observed data.

**Figure 16:**
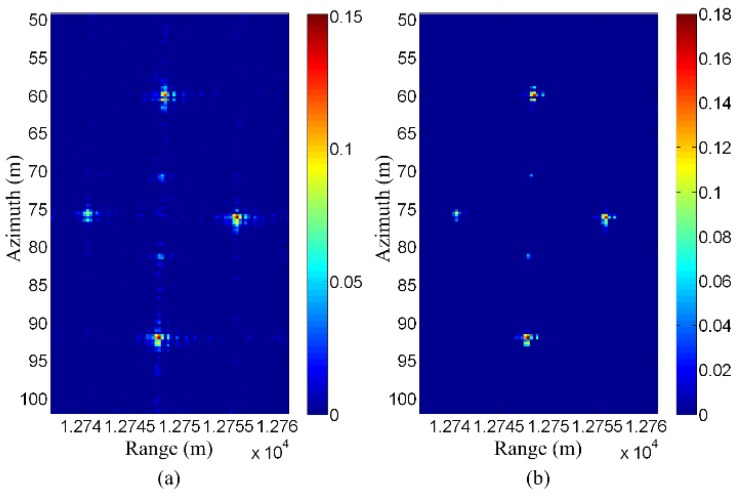
Observation position error compensation for airborne SAR data. (**a**) Result without observation position error compensation. (**b**) Result with observation position error compensation (taken from [84]).

Figure 16 shows a real data result using the method proposed in [84]. The data set used in this figure is the same as that used for Figure 9. In the data acquisition process, the airplane is expected to fly along a straight line. However, due to the air current’s influence, the trajectory of the airplane may slightly deviate from the expected one. As a result, the observation position data inevitably contain some errors.

Figure 16a shows the original CS reconstruction result. Since the observation position errors are not compensated, it can be seen that the targets are somewhat defocused. Figure 16b shows the corresponding CS reconstruction result with compensation for observation position error. It can be seen that the focusing quality is improved using the method proposed in [84]. The peak of the targets has an increase of about 20%, and the sidelobes are also significantly reduced.

## 4. Conclusions

In radar imaging area, there are many relevant techniques and methods, such as matched filtering, the range Doppler algorithm, the chirp scaling algorithm, the ωK algorithm, regularized methods, and CS methods. These techniques and methods are quite different in their forms. This paper tries to understand these techniques and methods in a unified mathematical framework.

Based on theoretical analysis, it can be seen that sparsity-driven regularization or CS-based radar imaging methods have potentially significant advantages. However, although many interesting ideas have been presented, very few of them have been verified with real data. There are still many unsolved or open problems in this area. In the issues discussed in this paper, the sampling scheme, fast reconstruction strategy, and model error problems are basically solved. However, issues concerning the sparsity or sparse representation of a complex and extended scene are still not completely solved. Strong clutter may break the sparsity of a scene, while sparse representation methods for an extended scene are currently not perfect. The state of the art in these areas has not yet reached the stage of practical application, and further investigations are needed in the future.

## Figures and Tables

**Table 1:** Characteristics and connections of radar imaging methods.

Radar Observation Model: s=Ag+n s: Observed Data, A: Measurement Matrix, g: Scene, n			
Imaging Methods	Mathematical Model	Characteristics	Equivalent Geometric Illustration
Least Squares (LS) Estimation	$\hat{g}=\arg\underset{g}{\min}\;{\left\|s\text{-}Ag\right\|}_{2}^{2}$	$\hat{g}={\left(A^{H}A\right)}^{-1}A^{H}s$, ${\left(A^{H}A\right)}^{-1}$ is usually ill-posed or nonexistent, cannot obtain a stable solution [9,13].	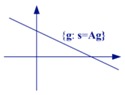
Matched Filtering	$\hat{g}=A^{H}s$	Avoids the ill-posed term in the LS solution, but the resolution is limited by the system bandwidth, and side-lobes will appear in the final image [4,21].	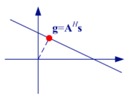
Range Doppler, Chirp Scaling, ωK, etc.	Approximations and transformations of $A^{H}s$	Approximations and transformations of the original matched filtering, in order to reduce the computational cost and make it more convenient to implement in practice [1].	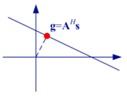
Regularization Method	$\hat{g}=\arg\underset{g}{\min}\;{\left\|s\text{-}Ag\right\|}_{2}^{2}+\lambda L(g)$	Add an extra constraint to the LS formula, so that the ill-posed inverse problem becomes well-posed. If the added constraint is chosen appropriately, the result will be better than that for matched filtering [8,9].	Depends on the expression of L(g).
Sparsity-Driven Regularization	$\hat{g}=\arg\underset{g}{\min}\;{\left\|s\text{-}Ag\right\|}_{2}^{2}+\lambda {\left\|g\right\|}_{p}^{p}$ 0≤p≤1	Choose L(g) as the $l_{p}$-norm (0≤p≤1), in order to obtain sparse reconstruction result [6,23].	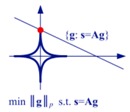
Bayesian MAP Estimation	$p(g)\propto \exp\left\{-\alpha {\left\|g\right\|}_{p}^{p}\right\}$ $\hat{g}=\arg\underset{g}{\min}\;{\left\|s\text{-}Ag\right\|}_{2}^{2}+2\sigma^{2}\alpha {\left\|g\right\|}_{p}^{p}$	For $2\sigma^{2}\alpha =\lambda$, the MAP estimation will be equivalent to the sparsity-driven regularization method [6,14].	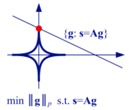
Compressed Sensing (CS) Method	$\hat{g}=\arg\underset{g}{\min}\;{\left\|g\right\|}_{0}\;s.t.\;{\left\|s\text{-}Ag\right\|}_{2}^{2}<\varepsilon$ or $\hat{g}=\arg\underset{g}{\min}\;{\left\|g\right\|}_{1}\;s.t.\;{\left\|s\text{-}Ag\right\|}_{2}^{2}<\varepsilon$	For an appropriate choice of λ, the CS method will be equivalent to the sparsity-driven regularization method [17,23].